# Atropia in Phthisical Sweating

**Published:** 1875-01

**Authors:** A. G. Smythe

**Affiliations:** Gun Town, Miss.


### ATROPIA IN PHTHISICAL SWEATING.

BY A. G. SMYTHE, M. D., GUN TOWN, MISS.

There is a short article in THE SOUTHERN MEDICAL RECORD for October, 1874, on page 616, entitled "Atropia in Phthisical Sweating." I have a case at this time in which the sweating became a great trouble, and having tried most of the usual remedies in such cases—all to no purpose—I had recourse to the extract of belladonna alone in a pill, in doses of the fourth of a grain, three times a day for three days. The sweating ceased immediately after the pupils manifested the influence of the medicine, and has not returned since, (twenty days). The only drawback in use of the extract was the checking of the pulmonary or bronchial secretions, which caused difficulty of breathing and expectoration, all of which subsided so soon as the pupils resumed their normal condition.

The question of a return of the sweating will be made a subject of further observation; also, if the extract is used again in the case, the action it may have upon the secretions and expectoration.

